# Use of the reverse shock index for identifying high-risk patients in a five-level triage system

**DOI:** 10.1186/s13049-016-0208-5

**Published:** 2016-02-09

**Authors:** Jung-Fang Chuang, Cheng-Shyuan Rau, Shao-Chun Wu, Hang-Tsung Liu, Shiun-Yuan Hsu, Hsiao-Yun Hsieh, Yi-Chun Chen, Ching-Hua Hsieh

**Affiliations:** Department of Trauma Surgery, Kaohsiung Chang Gung Memorial Hospital and Chang Gung University College of Medicine, No.123, Ta-Pei Road, Niao-Song District, Kaohsiung City, 833 Taiwan; Department of Neurosurgery, Kaohsiung Chang Gung Memorial Hospital and Chang Gung University College of Medicine, Kaohsiung City, Taiwan; Department of Anesthesiology, Kaohsiung Chang Gung Memorial Hospital and Chang Gung University College of Medicine, Kaohsiung City, Taiwan

**Keywords:** Reverse shock index (RSI), Shock, Injury severity score (ISS), Mortality, Five-level triage system

## Abstract

**Background:**

The ratio of systolic blood pressure (SBP) to heart rate (HR), called the reverse shock index (RSI), is used to evaluate the hemodynamic stability of trauma patients. To minimize undertriage in emergency departments (EDs), we evaluated whether RSI < 1 (i.e., SBP lower than HR) could be used as an additional variable to identify patients at high risk for more severe injury within a level category of the five-level Taiwan Triage and Acuity Scales (TTAS) system.

**Methods:**

Data obtained from the Trauma Registry System, including triage level according to the TTAS system, were retrospectively reviewed for trauma admissions from January 2009 through December 2013 in a Level I trauma center. In our study, the primary outcomes were injury severity as measured using different scoring systems, including the Glasgow coma scale (GCS), abbreviated injury scale scores, and the injury severity score (ISS), and in-hospital mortality. The secondary outcomes were hospital and intensive care unit (ICU) length of stay (LOS).

**Results:**

Of 10,814 trauma patients, 348 patients (3.2 %) had RSI < 1, whereas 10,466 (96.8 %) had RSI ≥ 1. Those with RSI < 1 had greater injury severity, a higher incidence of commonly associated injuries, lower GCS scores, greater deterioration of vital signs, and a higher incidence of procedures those with RSI ≥ 1. Patients with RSI < 1 also worse outcomes including hospital and ICU LOS, a greater frequency of ICU admission, and higher in-hospital mortality. Although the five-level TTAS system provides good prioritization of patients with major trauma, using the additional criterion of RSI < 1 could identify the patients at higher risk within the same triage level (I–III).

**Discussion:**

The alert of a trauma patient’s SBP being lower than his/her HR (RSI < 1) without the requirement of any additional equipment makes the concept of RSI particularly valuable in crowded EDs for identifying high-risk patients. RSI < 1 may serve as a principle trigger for action in the ED to alert trauma surgeons to the need for early intervention and timely preparation upon patient arrival particularly for those patients triaged in levels II and III of the TTAS system.

**Conclusions:**

RSI < 1 upon arrival at an ED is an alarming sign of an associated worse outcome. Within the same triage level from level I to level III, patients with RSI < 1 had worse outcomes than those with RSI ≥ 1. The inclusion of RSI in the TTAS system may help to identify patients with more serious injuries who need an upgraded management level.

## Background

In Taiwan, the five-level Taiwan Triage and Acuity Scales (TTAS) system was adapted from the Canadian Triage and Acuity Scales (CTAS) and was found to be a reliable triage system that accurately prioritizes the treatment of patients in the emergency department (ED) [1, 2]. The TTAS guidelines recommend a time to physician assessment based on the triage acuity level according to a classification of patients in descending order as follows: level I, resuscitation; level II, emergency; level III, urgent; level IV, less urgent; and level V, non-urgent [3]. It has been estimated that nearly one in three patients who experienced major trauma were undertriaged according to an analysis of 36,395 major trauma patients from the Nationwide Emergency Department Sample of the United States in 2010 [4]. Accurate triage of trauma patients is essential for trauma centers to avoid undertriage and poorer care or conversely overtriage and wasting of valuable resources and funding. To minimize undertriage, it is of the utmost importance to identify patients who are at high risk for severe injury; therefore, continuous evaluation of the triage system to provide greater acuity for use in emergency care is valuable for ensuring greater patient safety and more timely utilization of appropriate ED resources.

Hypovolemic shock is the most common type of shock in patients who experience traumatic injury. To identify hypovolemic shock, isolated vital signs such as heart rate (HR) or systolic blood pressure (SBP) have been revealed to be unreliable [5, 6]. Tachycardia failed to be useful as an isolated vital sign to predict the need for interventions commonly associated with hemorrhage control [6]. In addition, because there are compensatory mechanisms to increase cardiac output and maintain tissue perfusion despite a relatively low blood pressure, the reliance on SBP alone may delay recognition of the shock state [7]; for example, young patients who present with tachycardia and mild hypotension are at risk of losing their compensatory mechanisms, and therefore, they may slip into profound shock [7]. Furthermore, hemorrhagic shock occurs more rapidly in patients with penetrating injuries than in those who experience blunt trauma, and similar patterns regarding the cardiac index, mean arterial pressure, pulse oximetry, and transcutaneous oxygen tension indexed to FiO_2_ were found [8]. Moreover, different parameters other than vital signs, such as the Glasgow coma scale (GCS), are useful for triaging patients with head injuries [9]. By contrast, the shock index (SI), or the ratio of HR to SBP, has also been studied as a marker of significant injury in trauma patients with hypovolemic shock [10]. The SI upon ED arrival may be considered a clinical indicator of hypovolemic shock with respect to transfusion requirements and hemostatic resuscitation [11]. The SI has been previously emphasized to serve as a capable measure for assessing hemodynamic instability [11–15] and identifying patients requiring hospital admission and/or intensive care therapy despite vital signs that may not appear strikingly abnormal [16, 17]. When healthy blood donors were subjected to a defined blood loss of 450 mL, the SI substantially increased, whereas HR and SBP, taken as separate values, remained within their normal ranges [18]. It has been demonstrated that an abnormal SI portends a worse outcome in critically ill patients [15]. In addition, patients with SI ≥ 1.0 despite prehospital crystalloid resuscitation had significantly higher transfusion requirements and higher mortality than general major trauma patients [12].

Although the SI is an extremely practical and valuable predictor of outcome in trauma patients, the calculation of SI as the ratio of HR to SBP is odd and appears contradictory to the basic concept of shock. Generally, the idea of an unstable hemodynamic status is impressed upon practitioners as the patient’s SBP being lower than the HR but not the HR being higher than the SBP, as indicated by the SI. Therefore, we prefer to calculate the ratio of SBP to HR as the reverse shock index (RSI) to evaluate hemodynamic stability in trauma patients. RSI < 1 indicates that the SBP is lower than the HR, implying that the patient is probably in shock. Moreover, the RSI could be used upon the arrival of first responders at the site of injury without any additional calculation or equipment.

To minimize the chance of undertriage, many systems have adopted extensive lists of variables related to the mechanism of injury and patient demographics, but they often have little or no scientific validation. The objective of this study was to minimize the risk of undertriage in the ED by evaluating whether RSI < 1 as an additional variable can identify patients who are at high risk for more severe injury within a level category of the TTAS system.

## Methods

### Study design

This retrospective study was conducted at the Kaohsiung Chang Gung Memorial Hospital, a 2400-bed facility and Level I regional trauma center that provides care to trauma patients primarily from South Taiwan. This study reviewed all 16,548 hospitalized and registered patients added to the Trauma Registry System from January 1, 2009 to December 31, 2013. During this time, patients who were transferred from other hospitals were not included in the study population because their condition was generally stable after management or procedures had been performed in the previous hospital. In addition, patients who had incomplete data regarding vital signs, GCS, or triage level were excluded. In total, 10,814 trauma patients were enrolled in the study. Approval for this study was obtained from the hospital’s institutional review board (IRB) with approval numbers 103-4599B and 103-5679B before its initiation. The informed consent requirement was waived according to the IRB regulations.

Detailed patient information was retrieved from the Trauma Registry System of our institution, and it included data regarding triage level according to the TTAS system, age, sex, vital signs on arrival, GCS score assessed on arrival at the ED, details of procedures performed at the ED (intubation, chest tube insertion, and blood transfusion), abbreviated injury scale (AIS) scores for each body region, injury severity score (ISS), length of stay (LOS) in the hospital, LOS in the intensive care unit (ICU), in-hospital mortality, and complications associated with the injuries. The ISS is expressed as the median and interquartile range (IQR, Q_1_-Q_3_). The RSI was calculated as the ratio of SBP to HR (RSI = SBP/HR). In our study, the primary outcomes were injury severity as measured using different scoring systems (GCS, AIS, and ISS) and in-hospital mortality. The secondary outcomes were hospital LOS and ICU LOS. Odds ratios (ORs) were calculated with 95 % confidence intervals (CIs).

Data were compared using SPSS version 20 statistical software (IBM Corporation, Armonk, NY, USA). We used Pearson’s χ^2^ test, Fisher’s exact test, or the independent Student’s *t*-test, as applicable. All results are presented as means ± standard errors. A p-value of <0.05 was considered as statistically significant.

## Results

### Worse outcomes of patients with RSI < 1

**Fig. 1:**
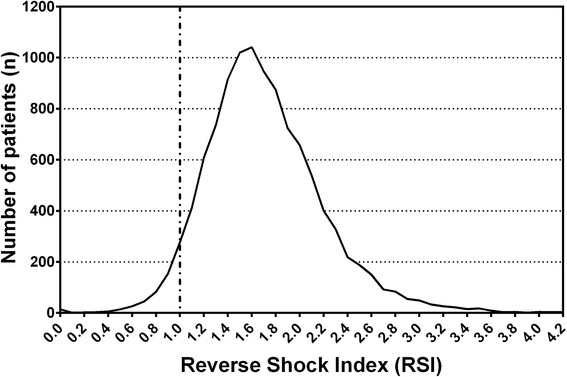
Distribution of trauma patients according to the reverse shock index (RSI)

**Fig. 2:**
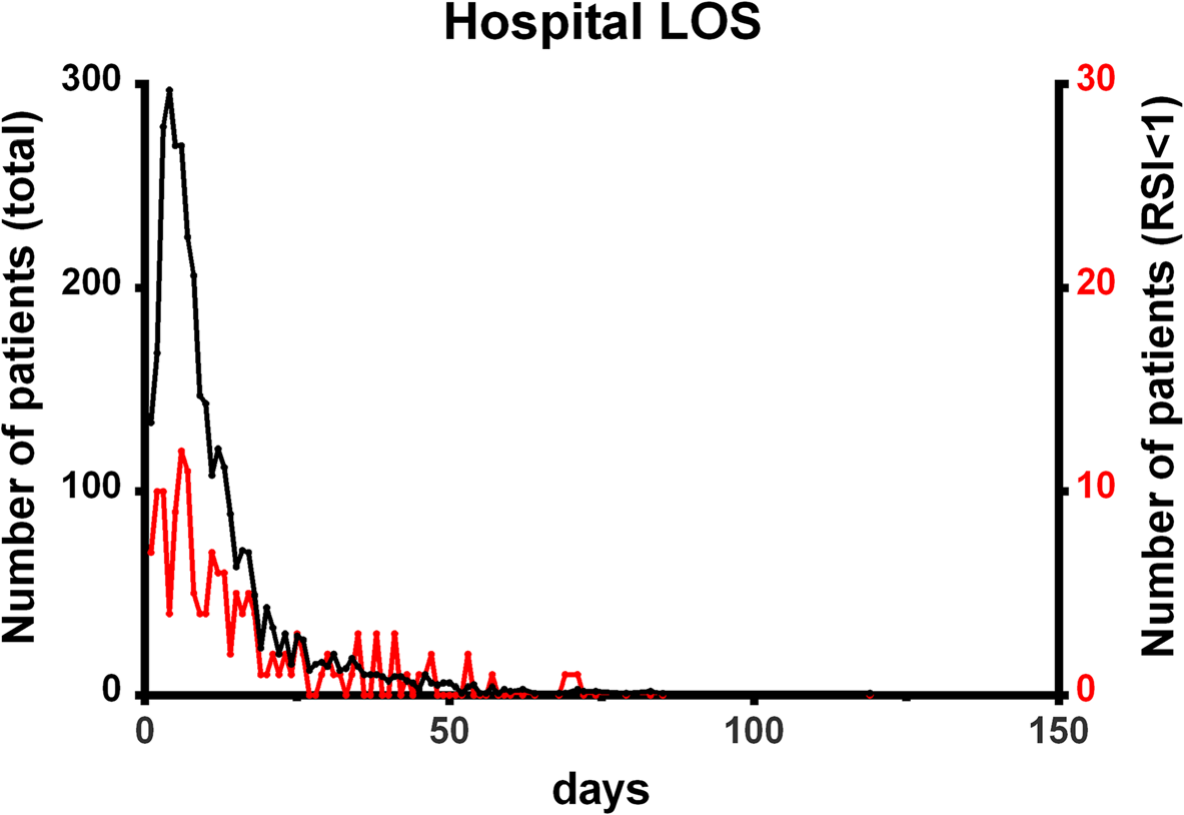
The hospital length of stay (LOS) of the trauma patients with reverse shock index (RSI) < 1 (red line) and RSI ≥ 1 (black line)

From January 1, 2009 to December 31, 2013, the Trauma Registry System included 16,548 hospitalized and registered patients. After excluding 5734 patients who were transferred from other hospitals or who had incomplete data, the study group included 10,814 patients. Of these patients, 348 patients (3.2 %) had RSI < 1, and 10,466 (96.8 %) had RSI ≥ 1 (Fig. 1). The mean RSIs of the patients with RSI < 1 and RSI ≥ 1 were 0.8 ± 0.2 and 1.7 ± 0.5, respectively (Table 1). A statistically significant difference regarding sex was found between patients with RSI < 1 (255 men [73.3 %] and 93 women [26.7 %]) and those with RSI ≥ 1 (6631 men [63.4 %] and 3835 women [36.6 %]). There were also significant differences in GCS scores and the distribution of scores (≤8, 9–12, or ≥13) between the RSI < 1 and RSI ≥ 1 groups. Analysis of AIS scores revealed that patients with RSI < 1 had significantly higher rates of injuries to the head/neck, face, thorax, and abdomen, whereas patients with RSI ≥ 1 displayed significantly higher rates of injuries to the extremities. Comparisons of trauma injury scores of the patients with RSI < 1 and those with RSI ≥ 1 revealed significant differences in the ISS between the groups (13 [5–22] vs. 5 [4–9]; p < 0.001) regardless of the stratification by injury severity (ISS of <16, 16–24, or ≥25). Likewise, we also found significant differences in in-hospital mortality rates between the two patient groups. The OR of mortality of the patients with RSI < 1 was 10.9-fold greater than that of patients with RSI ≥ 1 (95 % CI = 7.28–16.31). A significantly longer hospital LOS was found among patients with RSI < 1 than among those with RSI ≥ 1 (Fig. 2). Likewise, a significantly larger proportion of patients with RSI < 1 were admitted to the ICU, and the ICU LOS was significantly longer in this group. Furthermore, the patients with RSI < 1 exhibited a higher OR for presenting with worse hemodynamic measures than those with RSI ≥ 1 (Table 2). These measures included a GCS score < 13, SBP < 90 mmHg, HR > 100 beats/min, and a respiratory rate of <10 or >29 times/min. In addition, patients with RSI < 1 had higher odds for requiring procedures at the ED, including cardiopulmonary resuscitation, intubation, chest tube insertion, and blood transfusion. Patients with RSI < 1 had a statistically significantly higher OR for sustaining multiple types of trauma in the head, thorax, abdomen, and extremities (Table 3), although there were no significant differences regarding the rates of maxillofacial trauma and certain types of extremity trauma (humeral fracture and radial fractures).

**Table 1:** Demographics and injury characteristics of the hospitalized trauma patients

Variables	RSI < 1	RSI ≧ 1	*Odds ratio*	*p*
	*N* = 348	*N* = 10466	*(95 % CI)*	
SBP Mean (mmHg)	90.9±29.0	146.0±29.7	-	-
HR Mean (beats/min)	123.4±61.3	86.4±16.0	-	-
RSI Mean	0.8±0.2	1.7±0.5	-	-
Range	0.0−0.9	1.0−8.1	-	-
Gender				
Male	255 (73.3)	6631 (63.4)	1.6 (1.25–2.02)	<0.001
Female	93 (26.7)	3835 (36.6)	0.6 (0.50–0.80)	<0.001
GCS	12.1±4.0	14.4±2.0	–	<0.001
≤8	69 (19.8)	411 (3.9)	6.1 (4.57–8.02)	<0.001
9–12	36 (10.3)	338 (3.2)	3.5 (2.41–4.96)	<0.001
≥13	243 (69.8)	9717 (92.8)	0.2 (0.14–0.23)	<0.001
AIS, n (%)				
Head/Neck	139 (39.9)	2513 (24.0)	2.1 (1.69–2.62)	<0.001
Face	83 (23.9)	1946 (18.6)	1.4 (1.07–1.76)	0.013
Thorax	114 (32.8)	1197 (11.4)	3.8 (3.00–4.76)	<0.001
Abdomen	100 (28.7)	652 (6.2)	6.1 (4.75–7.76)	<0.001
Extremity	226 (64.9)	7590 (72.5)	0.7 (0.56–0.88)	0.002
ISS, median (IQR)	13 (5–22)	5 (4–9)	-	<0.001
<16	192 (55.2)	9152 (87.4)	0.2 (0.14–0.22)	<0.001
16–24	81 (23.3)	947 (9.0)	3.0 (2.36–3.95)	<0.001
≥25	75 (21.6)	367 (3.5)	7.6 (5.74–9.96)	<0.001
Mortality	34 (9.8)	103 (1.0)	10.9 (7.28–16.31)	<0.001
Ward LOS (days)	17.0±17.8	8.9±9.7	-	<0.001
ICU				
*n* (%)	168 (48.3)	1606 (15.3)	5.1 (4.15–6.40)	<0.001
LOS (days)	12.3±19.5	8.6±10.2	-	<0.001

**Table 2:** Physiological responses of and procedures underwent by the hospitalized trauma patients

Variables	RSI < 1	RSI ≧ 1	*Odds ratio*	*p*
	*N* = 348	*N* = 10466	*(95 % CI)*	
Physiological response, *n* (%)				
GCS < 13	105 (30.2)	749 (7.2)	5.6 (4.41–7.13)	<0.001
Respiratory rate <10 or >29	15 (4.3)	33 (0.3)	14.2 (7.66–26.47)	<0.001
Procedures performed, *n* (%)				
Cardiopulmonary resuscitation	12 (3.4)	11 (0.1)	33.9 (14.87–77.48)	<0.001
Intubation	82 (23.6)	369 (3.5)	8.4 (6.45–11.03)	<0.001
Chest tube insertion	36 (10.3)	172 (1.6)	6.9 (4.74–10.06)	<0.001
Blood transfusion	44 (12.6)	264 (2.5)	5.6 (3.99–7.85)	<0.001

**Table 3:** Associated injuries of the hospitalized trauma patients

Variables	RSI < 1	RSI ≧ 1	*Odds ratio*	*p*
	*N* = 348	*N* = 10466	*(95 % CI)*	
Head trauma, *n* (%)				
Cranial fracture	37 (10.6)	623 (6.0)	1.9 (1.32–2.67)	<0.001
Epidural hematoma (EDH)	22 (6.3)	401 (3.8)	1.7 (1.09–2.64)	0.018
Subdural hematoma (SDH)	38 (10.9)	770 (7.4)	1.5 (1.09–2.18)	0.013
Subarachnoid hemorrhage (SAH)	51 (14.7)	869 (8.3)	1.9 (1.40–2.57)	<0.001
Intracerebral hematoma (ICH)	14 (4.0)	169 (1.6)	2.6 (1.47–4.45)	0.001
Cervical vertebral fracture	11 (3.2)	91 (0.9)	3.7 (1.97–7.02)	<0.001
Maxillofacial trauma, *n* (%)				
Orbital fracture	3 (0.9)	226 (2.2)	0.4 (0.13–1.24)	0.098
Nasal fracture	6 (1.7)	120 (1.1)	1.5 (0.66–3.46)	0.323
Maxillary fracture	31 (8.9)	680 (6.5)	1.4 (0.97–2.05)	0.074
Mandibular fracture	14 (4.0)	265 (2.5)	1.6 (0.93–2.79)	0.084
Thoracic trauma, *n* (%)				
Rib fracture	56 (16.1)	833 (8.0)	2.2 (1.65–2.98)	<0.001
Sternal fracture	3 (0.9)	14 (0.1)	6.5 (1.86–22.69)	0.001
Hemothorax	20 (5.7)	150 (1.4)	4.2 (2.60–6.77)	<0.001
Pneumothorax	16 (4.6)	163 (1.6)	3.0 (1.80–5.15)	<0.001
Hemopneumothorax	30 (8.6)	125 (1.2)	7.8 (5.16–11.81)	<0.001
Lung contusion	15 (4.3)	111 (1.1)	4.2 (2.42–7.28)	<0.001
Thoracic vertebral fracture	14 (4.0)	93 (0.9)	4.7 (2.64–8.29)	<0.001
Abdominal trauma, n (%)				
Intra-abdominal injury	18 (5.2)	126 (1.2)	4.5 (2.70–7.42)	<0.001
Hepatic injury	42 (12.1)	182 (1.7)	7.8 (5.44–11.05)	<0.001
Splenic injury	26 (7.5)	87 (0.8)	9.6 (6.13–15.14)	<0.001
Retroperitoneal injury	10 (2.9)	12 (0.1)	25.8 (11.06–60.07)	<0.001
Renal injury	12 (3.4)	45 (0.4)	8.3 (4.34–15.78)	<0.001
Urinary bladder injury	3 (0.9)	17 (0.2)	5.3 (1.56–18.32)	0.003
Lumbar vertebral fracture	14 (4.0)	179 (1.7)	2.4 (1.38–4.20)	0.001
Sacral vertebral fracture	7 (2.0)	62 (0.6)	3.4 (1.57–7.58)	0.001
Extremity trauma, n (%)				
Scapular fracture	13 (3.7)	160 (1.5)	2.5 (1.41–4.45)	0.001
Humeral fracture	15 (4.3)	468 (4.5)	1.0 (0.57–1.63)	0.886
Radial fracture	28 (8.0)	1069 (10.2)	0.8 (0.52–1.14)	0.188
Ulnar fracture	28 (8.0)	514 (4.9)	1.7 (1.14–2.52)	0.008
Pelvic fracture	44 (12.6)	269 (2.6)	5.5 (3.91–7.70)	<0.001
Femoral fracture	57 (16.4)	842 (8.0)	2.2 (1.67–3.00)	<0.001
Tibial fracture	37 (10.6)	768 (7.3)	1.5 (1.06–2.13)	0.021
Fibular fracture	26 (7.5)	425 (4.1)	1.9 (1.26–2.88)	0.002

### Good prioritization of patients using the TTAS system

The overall study population of 10,814 trauma patients was triaged into levels I (623 patients), II (3333 patients), III (6522 patients), IV (314 patients), and V (22 patients). The comparative injury characteristics between levels I and II as well as between levels II and III are shown in Table 4. Differences between sexes were found between levels II and III (p < 0.001) but not between levels I and II (*p* = 0.173). Good prioritization using the five-level TTAS system was found with significant differences among patients with major trauma (levels I–III) regarding ISS, hospital LOS, the proportion of patients admitted to the ICU, ICU LOS, and mortality.

**Table 4:** Comparison of the trauma patients regarding sex and outcomes in levels I–III in the Taiwan Triage and Acuity Scales system

Variables	Triage								
	I	II			III			IV	V
	*N* = 623	*N* = 3333	I vs. II		*N* = 6522	II vs. III		*N* = 314	*N* = 22
			*Odds ratio (95 % CI)*	*p*		*Odds ratio (95 % CI)*	*p*		
Gender				0.173			<0.001		
Male (n, %)	455 (73.0)	2344 (70.3)	1.1 (0.94–1.38)		3899 (59.8)	1.6 (1.46–1.74)		175 (55.7)	13 (59.1)
Female (n, %)	168 (27.0)	989 (29.7)	0.9 (0.72–1.06)		2623 (40.2)	0.6 (0.57–0.69)		139 (44.3)	9 (40.9)
ISS									
Median (IQR)	17 (10–25)	8 (4–13)		<0.001	4 (4–9)		<0.001	4 (4–9)	4 (4–9)
LOS (days)	19.3±19.8	11.3±11.1		<0.001	7.3±7.3		<0.001	7.2±7.1	9.0±8.5
ICU									
n (%)	416 (66.8)	809 (24.3)	6.3 (5.21–7.54)	<0.001	499 (7.7)	3.9 (3.43–4.37)	<0.001	47 (15.0)	3 (13.6)
Days	12.5±14.9	8.3±11.3		<0.001	7.2±7.1		0.040	8.1±8.7	4±2.6
Mortality	96 (15.4)	27 (0.8)	22.3 (14.4–34.5)	<0.001	14 (0.2)	3.8 (1.99–7.25)	<0.001	0 (0.0)	0 (0.0)

### Patients with RSI < 1 within levels I–III had worse outcomes

We compared patients with RSI < 1 and those with RSI ≥ 1 in levels I–III (Table 5). In level I, there were 130 patients with RSI < 1 and 493 patients with RSI ≥ 1. No sex-related differences were found in this group. The patients with RSI < 1 had worse outcomes than those with RSI ≥ 1 regarding ISS and ICU LOS but not hospital LOS, the proportion of patients admitted to the ICU, or mortality. In level II, there were 153 patients with RSI < 1 and 3180 patients with RSI ≥ 1. A higher proportion of male patients had RSI < 1 in this level. The patients with RSI < 1 had worse outcome than those with RSI ≥ 1 regarding ISS, hospital LOS, the proportion of patients admitted to the ICU, and mortality but not ICU LOS. In level III, there were 59 patients with RSI < 1 and 6463 patients with RSI ≥ 1. No sex-related differences were found in this group. The patients with RSI < 1 had worse outcomes than those with RSI ≥ 1 regarding ISS, hospital LOS, the proportion of patients admitted to the ICU, and mortality but not ICU LOS.

**Table 5:** Sex and outcomes of the trauma patients with reverse shock index (RSI) < 1 or RSI ≥ 1 in levels I–III of the Taiwan Triage and Acuity Scales system

Triage: I	RSI < 1	RSI ≧ 1	*Odds ratio*	*p*
Variables	*N* = 130	*N* = 493	*(95 % CI)*	
Gender				0.990
Male (*n*, %)	95 (73.1)	360 (73.0)	1.0 (0.65–1.55)	
Female (*n*, %)	35 (26.9)	133 (27.0)	1.0 (0.65–1.54)	
ISS				
Median (IQR)	20 (12–29)	17 (9–25)	-	0.007
LOS days	21.8±22.9	18.7±18.8	-	0.102
ICU				
*n* (%)	92 (70.8)	324 (65.7)	1.3 (0.83–1.92)	0.277
Days	15.5±22.9	11.7±11.5	-	0.030
Mortality	26 (20.0)	70 (14.2)	1.5 (0.92–2.49)	0.105
Triage: II	RSI < 1	RSI ≧ 1	*Odds ratio*	*p*
Variables	*N* = 153	*N* = 3180	*(95 % CI)*	
Gender				0.025
Male (n, %)	120 (78.4)	2224 (69.9)	1.6 (1.06–2.32)	
Female (n, %)	33 (21.6)	956 (30.1)	0.6 (0.43–0.95)	
ISS				
Median (IQR)	10 (4–17)	6 (4–13)	-	<0.001
LOS days	15.0±14.3	11.1±10.9	-	<0.001
ICU				
*n* (%)	61 (39.9)	748 (23.5)	2.2 (1.54–3.01)	<0.001
days	8.8±14.8	8.2±11.0	-	0.698
Mortality	7 (4.6)	20 (0.6)	7.2 (2.98–17.31)	<0.001
Triage III	RSI < 1	RSI ≧ 1	*Odds ratio*	*p*
Variables	*N* = 59	*N* = 6463	*(95 % CI)*	
Gender				0.846
Male (*n*, %)	36 (61.0)	3863 (59.8)	1.1 (0.62–1.78)	
Female (*n*, %)	23 (39.0)	2600 (40.2)	0.9 (0.56–1.61)	
ISS				
Median (IQR)	5 (4–16)	4 (4–9)	-	<0.001
LOS days	12.2±10.4	7.2±7.2	-	<0.001
ICU				
*n* (%)	15 (25.4)	484 (7.5)	4.2 (2.33–7.62)	<0.001
Days	7.0±6.9	7.2±7.2	-	0.932
Mortality	1 (1.7)	13 (0.2)	8.5 (1.09–66.15)	0.041

**Table 6:** (RSI < 1). Comparison of the trauma patients with reverse shock index < 1 in levels I–III in the Taiwan Triage and Acuity Scales system

Variables	Triage								
	I	II			III			IV	V
	*N* = 130	*N* = 153	I vs. II		*N* = 59	II vs. III		*N* = 5	*N* = 1
			*Odds ratio (95 % CI)*	*p*		*Odds ratio (95 % CI)*	*p*		
Gender				0.293			0.010		
Male (n, %)	95 (73.1)	120 (78.4)	0.7 (0.43–1.29)		36 (61.0)	2.3 (1.21–4.45)		3 (60.0)	1 (100)
Female (n, %)	35 (26.9)	33 (21.6)	1.3 (0.78–2.31)		23 (39.0)	0.4 (0.23–0.82)		2 (40.0)	0 (0)
ISS									
Median (IQR)	20 (12–29)	10 (4–17)		<0.001	5 (4–16)		0.098	5 (3–14)	10 (10–10)
LOS (days)	21.8±22.9	15.0±14.3		0.002	12.2±10.4		0.169	4.3±9.6	11-
ICU									
*n* (%)	92 (70.8)	61 (39.9)	3.7 (2.22–6.01)	<0.001	15 (25.4)	1.9 (1.00–3.80)	0.051	0 (0)	0 (0)
Days	15.5±22.9	8.8±14.8		0.046	7.0±6.9		0.648	--	--
Mortality	26 (20.0)	7 (4.6)	5.2 (2.18–12.47)	<0.001	1 (1.7)	2.8 (0.34–23.10)	0.324	0 (0)	0 (0)

### Inaccurate prioritization of patients with RSI < 1 within levels II and III in the TTAS system

As shown in Table  6, the 348 trauma patients with RSI < 1 were triaged into levels I (130 patients), II (153 patients), III (59 patients), IV (5 patients), and V (1 patient). The comparative injury characteristics between levels I and II as well as between levels II and III are shown. Sex-related differences were found between levels II and III (*p* = 0.010) but not between levels I and II (*p* = 0.293). Good prioritization of trauma patients with RSI < 1 using the TTAS system was achieved between levels I and II but not between levels II and III. The difference between levels I and II was significant concerning ISS, hospital LOS, the proportion of patients admitted to the ICU, ICU LOS, and mortality. However, none of these outcome measurements was significantly different between levels II and III, implying there was an inaccurate prioritization between levels II and III for patients with RSI < 1.

### Inaccurate prioritization of patients with RSI < 1 within levels II and III in the TTAS system

To investigate the outcomes of patients with SBP < 90 who were inappropriately triaged to a level other than level I in the TTAS system, further stratification of 153 patients with RSI < 1 in level II or III using the criterion SBP < 90 was performed. We found that in level III, no patient had SBP < 90. In triage level II, there were 67 patients with RSI < 1 and SBP < 90 and 86 patients with RSI < 1 and SBP ≥ 90 (Table 7). Notably, these nonhypotensive patients with RSI < 1 who were triaged in level II displayed significant differences from those with RSI ≥ 1 concerning ISS, hospital LOS, the proportion of patients admitted to the ICU, and mortality, indicating the RSI < 1 is useful for identifying high-risk patients triaged in level II who displayed a nonhypotensive status.

**Table 7:** Comparison of trauma patients with reverse shock index (RSI) < 1 and systolic blood pressure ≥ 90 with those with RSI ≥ 1 in level II

Triage II	RSI < 1	RSI > 1	*Odds ratio*	*p*
Variables	SBP ≧ 90	*n* = 3180	*(95 % CI)*	
	*n* = 86			
Gender				
Male (n, %)	72 (83.7)	2224 (69.9)	2.2 (1.24–3.94)	0.006
Female (n, %)	14 (16.3)	956 (30.1)	0.5 (0.25–0.81)	0.006
ISS				
Median (IQR)	10 (4–18)	6 (4–13)	-	<0.001
LOS				
Days	15.8±15.3	11.1±10.9	-	0.006
LOS in ICU				
*n* (%)	36 (41.9)	748 (23.5)	2.3 (1.51–3.62)	<0.001
Days	10.3±18.4	8.2±11.0	-	0.499
Mortality	10 (11.6)	20 (0.6)	20.8 (9.41–45.92)	<0.001

## Discussion

The assessment and treatment of trauma patients upon arrival to the ED is essential in the presence of life-threatening injuries. Prospectively identifying patients who would benefit from trauma care is essential to the success of trauma systems. However, this remains an ongoing challenge for prehospital providers and the physicians in attendance in the emergency room, who have limited data to make the decision. Moreover, this decision has been clearly illustrated to have implications for patient outcomes [19]. In this study, we analyzed the injury characteristics of all trauma patients hospitalized in a Level I trauma center and found that patients who presented with RSI < 1 had a higher injury severity, a higher incidence of commonly associated injuries, a worse physiological response, and a greater frequency of undergoing procedures than those with RSI ≥ 1. Furthermore, patients with RSI < 1 also had worse outcomes including hospital and ICU LOS, the proportion of patients admitted to the ICU, and in-hospital mortality. Moreover, although we found that the five-level TTAS system could provide good prioritization of patients with major trauma, within the same triage level from level I to level III, patients with RSI < 1 had worse outcomes than those with RSI ≥ 1.

Under the circumstance of many patients waiting in crowded EDs for hours before being evaluated [20], triage tools are expected to have high sensitivity in discriminating emergency conditions in terms of risk management for the care of trauma patients. However, it is also important for triage systems to balance both patient safety and system efficiency. In a study of the reproducibility of the five-level CTAS, the overall interrater agreement was moderate among five experienced nurses, with a global κ of 0.44 (95 % *CI* = 0.40–0.48) [21]. It has been reported that through years of practice, nurses might interpret and integrate the CTAS differently, developing an individualized usage of the tool, which also could lead to lower interrater reliability [21]. The diversity among several aspects of nursing triage may point to a safety risk for patients. One of the major benefits of using the RSI for evaluation at the ED is that it can be used quickly when first responders arrive without the requirement of any additional equipment or cost. Following the advanced trauma life support paradigm of “keep algorithms simple,” RSI < 1 may serve as a principle trigger for action in the ED. RSI < 1 also can alert trauma surgeons to the need for early intervention and timely preparation upon patient arrival. In this study, the OR for mortality of the entire trauma patient population with RSI < 1 was more than 10-fold greater than that of patients with RSI ≥ 1. In addition, the ORs for mortality of the patients with RSI < 1 were approximately 8.5- and 7.2-fold greater than those for patients with RSI ≥ 1 in triage levels III and II, respectively. The inclusion of the RSI in the TTAS triage system may help to identify patients with serious injuries who need an upgraded management level and avert morbidity or mortality after a severe injury. Even if we are unable to identify reductions of morbidity and mortality after addition of the RSI in the TTAS triage system, we may conservatively assume they exist. However, a prospective study is warranted to validate the aforementioned hypothesis.

In this study, good prioritization of the five-level TTAS system is found with significant differences among patients with major trauma (levels I–III). However, inaccurate prioritization in levels II and III of the TTAS system was found when the patients had RSI < 1. The middle group (level III) is most problematic because it includes the greatest number of patients, but this group had a relatively low mortality rate. These findings may result in physicians not paying close attention to these patients to avoid using limited resources that could be reserved for other potentially sicker patients, which that may make the physician less alert and make the limited resources being taken away from other potentially sicker patients [22]. The original recommendation of those endorsing the TTAS was that level III patients should be evaluated by a physician within 30–60 min. This recommendation is likely to be safe for these patients. Unfortunately, there are instances in which level III patients may wait for hours in a crowded ED before they are actually placed in a treatment area and evaluated by a physician. In this study, level III comprised 60 % of all patients, but this group had a low mortality rate (0.2 %). If these higher-risk level III patients are reassigned to level II by delineating the high-risk group of patients using RSI < 1 as an additional criterion, one would expect that placing these patients in a higher triage category may reduce delays in their evaluation and treatment and subsequently reduce the morbidity associated with such delays. Although different parameters such as age [23, 24], GCS [9], injury mechanisms [25], and injury regions [26] appear to be outcome predictors of trauma patients irrespective of vital signs, the alert of a trauma patient’s SBP being lower than his/her HR (RSI < 1) without the requirement of any additional equipment makes the concept of RSI particularly valuable in crowded EDs for identifying high-risk patients.

Our analysis has several limitations. First, our data were collected prospectively as part of the required trauma registry process, but our questionnaires and analyses were performed retrospectively, making them subject to the limitations of all retrospective studies. Second, injured patients who died prior to arrival at the hospital or who were discharged from the ED were not included in the sample, which could result in bias. Third, the impact of pre-existing comorbidities on the course of hospitalization and mortality remains unclear. In addition, the lack of available data regarding the circumstances of the injury and the factors influencing the decision-making regarding patient management may have biased the outcome, particularly because the study population was limited to a single urban trauma center. Last, some important data other than LOS and mortality, including costs, delays in treatment, and complications, were not evaluated, and these data may provide limited information concerning the outcome evaluation.

## Conclusion

Our analysis of data on trauma admissions at a Level I trauma center spanning 5 years indicated that patients with RSI < 1 had worse outcomes including hospital and ICU LOS, the proportion of patients admitted to the ICU, and in-hospital mortality. Although the five-level TTAS system could provide good prioritization of patients with major trauma, within the same triage level (I–III), the patients with RSI < 1 had worse outcomes than those with RSI ≥ 1. The inclusion of the RSI in the TTAS triage system may help to identify the patients with serious injuries who need to be shifted to a higher triage category.

## Abbreviations

ORs: Odd ratios
AIS: Abbreviated injury scale
CI: Confidence intervals
GCS: Glasgow coma scale
HR: Heart rate
ICU: Intensive care unit
ISS: Injury severity score
LOS: Length of stay
RSI: Reverse shock index
SI: Shock index
SBP: Systolic blood pressure
TTAS: Taiwan Triage and Acuity Scales

## References

[1] Ng CJ, Yen ZS, Tsai JC, Chen LC, Lin SJ, Sang YY (2011). Validation of the Taiwan triage and acuity scale: a new computerised five-level triage system. Emerg Med J.

[2] Chang YC, Ng CJ, Wu CT, Chen LC, Chen JC, Hsu KH (2013). Effectiveness of a five-level Paediatric Triage System: an analysis of resource utilisation in the emergency department in Taiwan. Emerg Med J.

[3] Ng CJ, Hsu KH, Kuan JT, Chiu TF, Chen WK, Lin HJ (2010). Comparison between Canadian Triage and Acuity Scale and Taiwan Triage System in emergency departments. J Formos Med Assoc.

[4] Xiang H, Wheeler KK, Groner JI, Shi J, Haley KJ (2014). Undertriage of major trauma patients in the US emergency departments. Am J Emerg Med.

[5] Victorino GP, Battistella FD, Wisner DH (2003). Does tachycardia correlate with hypotension after trauma?. J Am Coll Surg.

[6] Brasel KJ, Guse C, Gentilello LM, Nirula R (2007). Heart rate: is it truly a vital sign?. J Trauma.

[7] Gutierrez G, Reines HD, Wulf-Gutierrez ME (2004). Clinical review: hemorrhagic shock. Crit Care.

[8] Lu KJ, Chien LC, Wo CC, Demetriades D, Shoemaker WC (2006). Hemodynamic patterns of blunt and penetrating injuries. J Am Coll Surg.

[9] Matsuyama T, Okuchi K, Seki T, Murao Y (2004). Prognostic factors in hanging injuries. Am J Emerg Med.

[10] King RW, Plewa MC, Buderer NM, Knotts FB (1996). Shock index as a marker for significant injury in trauma patients. Acad Emerg Med.

[11] Mutschler M, Nienaber U, Munzberg M, Wolfl C, Schoechl H, Paffrath T (2013). The Shock Index revisited - a fast guide to transfusion requirement? A retrospective analysis on 21,853 patients derived from the TraumaRegister DGU. Crit Care.

[12] Mitra B, Fitzgerald M, Chan J (2014). The utility of a shock index >/= 1 as an indication for pre-hospital oxygen carrier administration in major trauma. Injury.

[13] McNab A, Burns B, Bhullar I, Chesire D, Kerwin A (2013). An analysis of shock index as a correlate for outcomes in trauma by age group. Surgery.

[14] DeMuro JP, Simmons S, Jax J, Gianelli SM (2013). Application of the Shock Index to the prediction of need for hemostasis intervention. Am J Emerg Med..

[15] Cannon CM, Braxton CC, Kling-Smith M, Mahnken JD, Carlton E, Moncure M (2009). Utility of the shock index in predicting mortality in traumatically injured patients. J Trauma.

[16] Yealy DM, Delbridge TR (1994). The shock index: all that glitters. Ann Emerg Med.

[17] Rady MY, Smithline HA, Blake H, Nowak R, Rivers E (1994). A comparison of the shock index and conventional vital signs to identify acute, critical illness in the emergency department. Ann Emerg Med.

[18] Birkhahn RH, Gaeta TJ, Terry D, Bove JJ, Tloczkowski J (2005). Shock index in diagnosing early acute hypovolemia. Am J Emerg Med..

[19] MacKenzie EJ, Rivara FP, Jurkovich GJ, Nathens AB, Frey KP, Egleston BL (2006). A national evaluation of the effect of trauma-center care on mortality. N Engl J Med.

[20] Travers DA, Waller AE, Bowling JM, Flowers D, Tintinalli J (2002). Five-level triage system more effective than three-level in tertiary emergency department. J Emerg Nurs.

[21] Dallaire C, Poitras J, Aubin K, Lavoie A, Moore L (2012). Emergency department triage: do experienced nurses agree on triage scores?. J Emerg Med.

[22] Ruger JP, Lewis LM, Richter CJ (2007). Identifying high-risk patients for triage and resource allocation in the ED. Am J Emerg Med.

[23] Bruijns SR, Guly HR, Bouamra O, Lecky F, Lee WA (2013). The value of traditional vital signs, shock index, and age-based markers in predicting trauma mortality. J Trauma Acute Care Surg..

[24] Zarzaur BL, Croce MA, Magnotti LJ, Fabian TC (2010). Identifying life-threatening shock in the older injured patient: an analysis of the National Trauma Data Bank. J Trauma.

[25] Haider AH, Chang DC, Haut ER, Cornwell EE, Efron DT (2009). Mechanism of injury predicts patient mortality and impairment after blunt trauma. J Surg Res.

[26] Van Belleghem G, Devos S, De Wit L, Hubloue I, Lauwaert D, Pien K (2016). Predicting in-hospital mortality of traffic victims: A comparison between AIS-and ICD-9-CM-related injury severity scales when only ICD-9-CM is reported. Injury.

